# The Modified Alar Batten Graft as a Tip-Alar Superstructure: A Technique to Restore Soft Triangle Stability and External Valve Support

**DOI:** 10.7759/cureus.112002

**Published:** 2026-07-03

**Authors:** Alfonso Reyes Escobedo, Oscar Ruben Santos Santos, Javier González Reyes, Berenice Tamayo Garcia, David Salvador Dorado Martinez, Paola Hernandez Rosales

**Affiliations:** 1 Otolaryngology - Head and Neck Surgery, Concept Clinic, San Pedro Garza Garcia, MEX; 2 General Surgery, Hospital Christus Muguerza Alta Especialidad, Monterrey, MEX

**Keywords:** alar batten graft, external nasal valve collapse, lateral nasal wall insufficiency, nasal tip support, rhinoplasty, soft triangle

## Abstract

Lateral nasal wall insufficiency and external nasal valve collapse are frequent causes of both functional impairment and aesthetic deformity following primary or previous nasal surgery. Traditional alar batten grafts provide structural reinforcement but have a limited impact on alar margin contour and tip-alar harmony. We present a modification of the classic batten graft, placed over the lateral crura as a superstructure following tip framework construction during open rhinoplasty, through an illustrative case. The graft is carved in a trapezoidal configuration and secured at the domal level, allowing controlled modulation of tip contour and external valve support. The modified graft provided structural reinforcement of the lateral nasal wall and improved nasal tip aesthetics. Graft placement stabilized the soft triangle and restored continuity of the alar-tip unit by improving alar retraction and tip definition. Postoperatively, the patient reported a Nasal Obstruction Symptom Evaluation (NOSE) score of 5 and a Rhinoplasty Outcome Evaluation (ROE) score of 87.5, with no reported complications. The modified batten graft is a versatile technique that addresses both the functional and aesthetic components of zone II lateral nasal wall insufficiency. It represents a useful option for selected patients requiring simultaneous external valve support and refinement of nasal tip contour.

## Introduction

In functional rhinoplasty, alar batten grafts are a well-established approach for managing internal and external nasal valve collapse [1]. The technique consists of placing a curved cartilage graft within a pocket at the site of maximum insufficiency [1]. Nasal surgery, particularly aggressive resection of the lateral crura, is a frequent cause of external nasal valve collapse [2]. Zone 1 of the nose corresponds to the dynamic movement of the upper lateral cartilages and the scroll area, whereas zone 2 encompasses the lower lateral cartilages and alar soft tissues, collectively described as the external nasal valve [3]. Distortion of the lateral nasal wall components may affect nasal tip and alar harmony while compromising lateral nasal wall support [4].

Traditional alar batten grafts provide structural reinforcement but have a limited impact on tip-alar harmony. In this article, we present a modification of the classic batten graft, placed over the lower lateral crura in zone 2 as a superstructure following tip framework construction. The surgical technique is described in detail and illustrated through a selected case involving a weak lateral nasal wall and external nasal valve collapse, with opportunities for aesthetic improvement of the alar margins and nasal tip. The graft is intended to provide structural support to the lateral nasal wall while restoring continuity of the tip-alar complex and improving alar margin contour, nasal tip definition, and soft triangle stability.

## Technical report

Graft material

For this technique, septal cartilage is preferred for its availability and ease of shaping, with costal cartilage as a suitable alternative when septal cartilage is insufficient.

Timing within the operation and the “superstructure” concept

After completing the desired tip framework using the surgeon's preferred technique, the modified batten grafts are placed as a superstructure over the reconstructed tip complex.

Graft design and preparation

**Figure 1:**
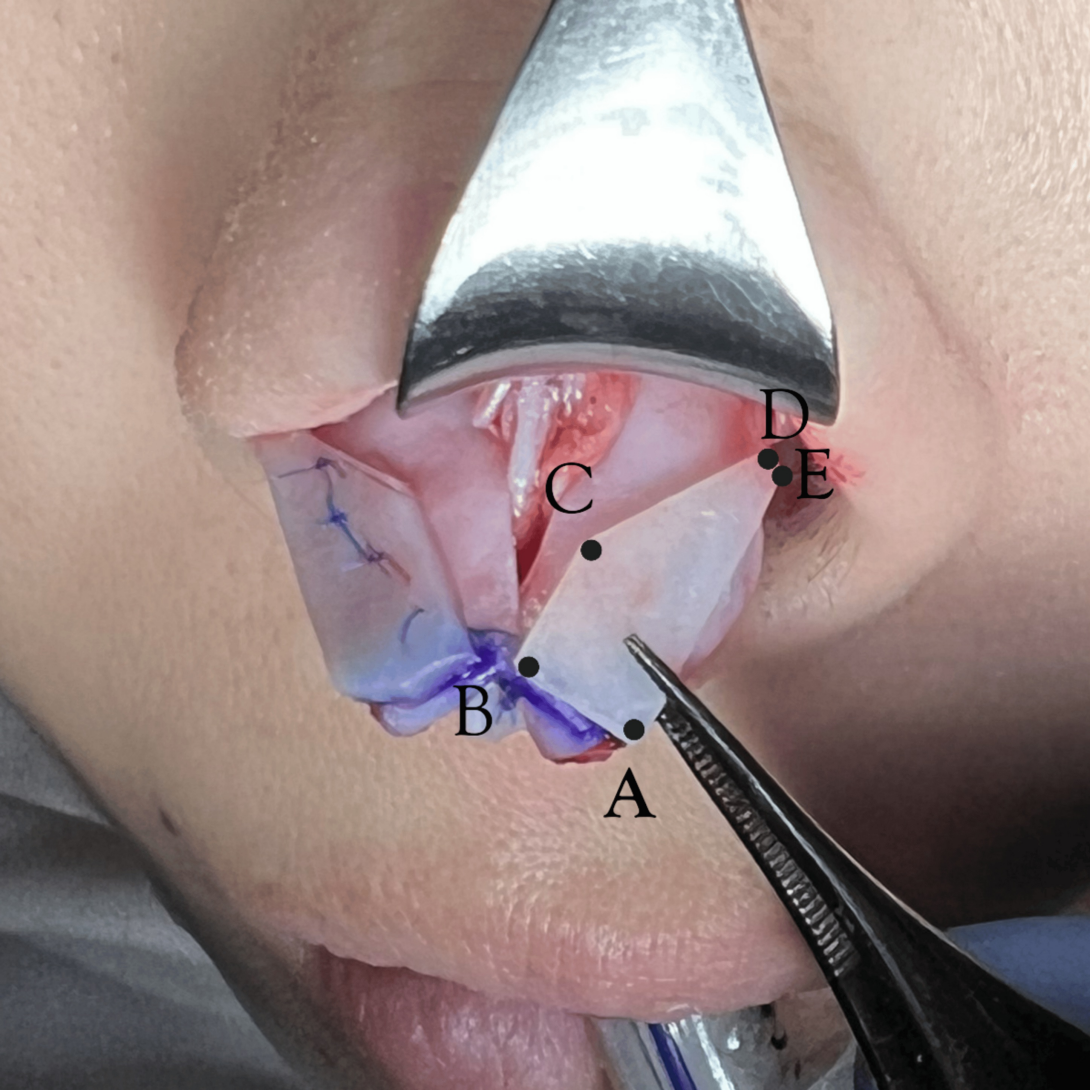
Trapezoidal graft design and dimensions A-E: long axis (15 mm). A-B: width (5 mm). B-C: contralateral length (5 mm). C-D: final connecting cut.

Each graft is carved in a trapezoidal configuration and secured at the domal level, allowing controlled modulation of the tip and alar margins while providing external valve support. The dimensions are adapted to the patient's lateral crura. As a standard reference, the graft measures approximately 15 mm along its long axis (A-E), 5 mm in width (A-B), and 5 mm along the opposite edge (B-C), with a final cut (C-D) connecting this edge to the long axis (Figure 1).

Vestibular mucosal dissection

**Figure 2:**
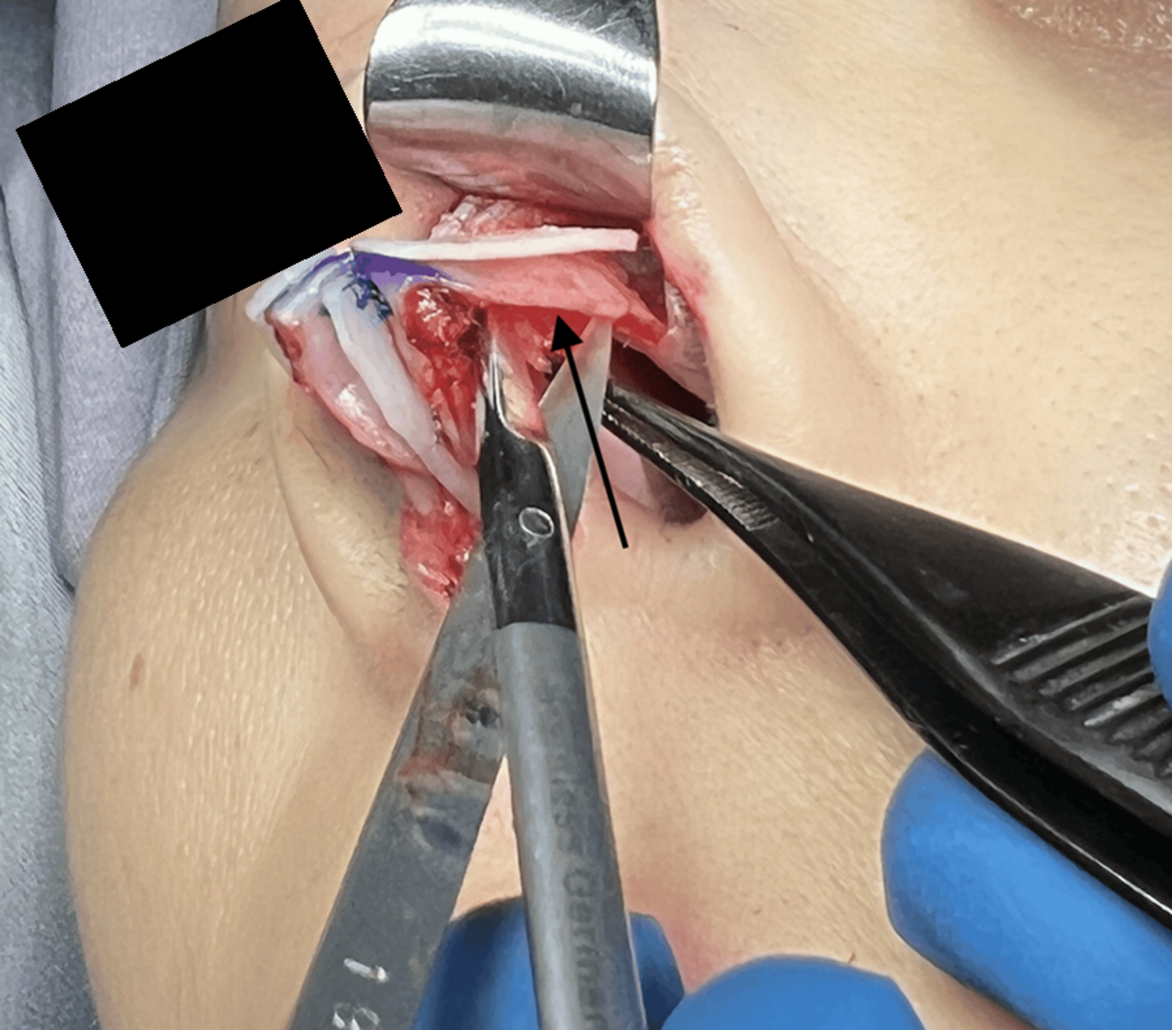
Dissection plane The black arrow points to the vestibular mucosal dissection plane.

Fixation requires careful elevation of the vestibular mucosa from the lateral crus. The area is infiltrated to facilitate hydrodissection, followed by meticulous dissection with fine scissors to elevate the vestibular mucosa from the cartilaginous surface of the lateral crus (Figure 2). 

Graft positioning and fixation

**Figure 3:**
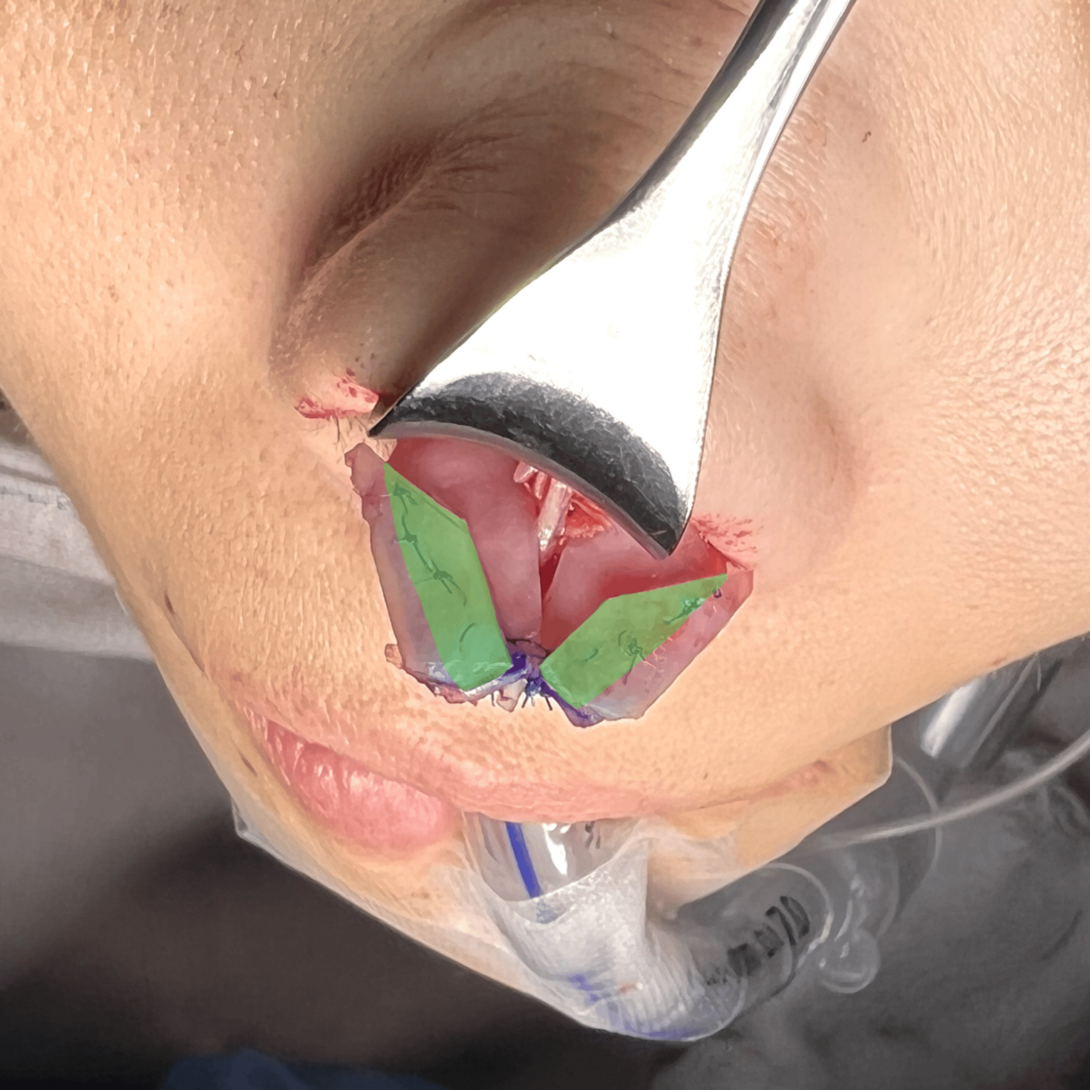
Cephalic view of the graft The area highlighted in green represents the modified alar batten graft support area over the lower lateral cartilages. The lateral portion of the graft corresponds to the additional margin that the superstructure provides.

**Figure 4:**
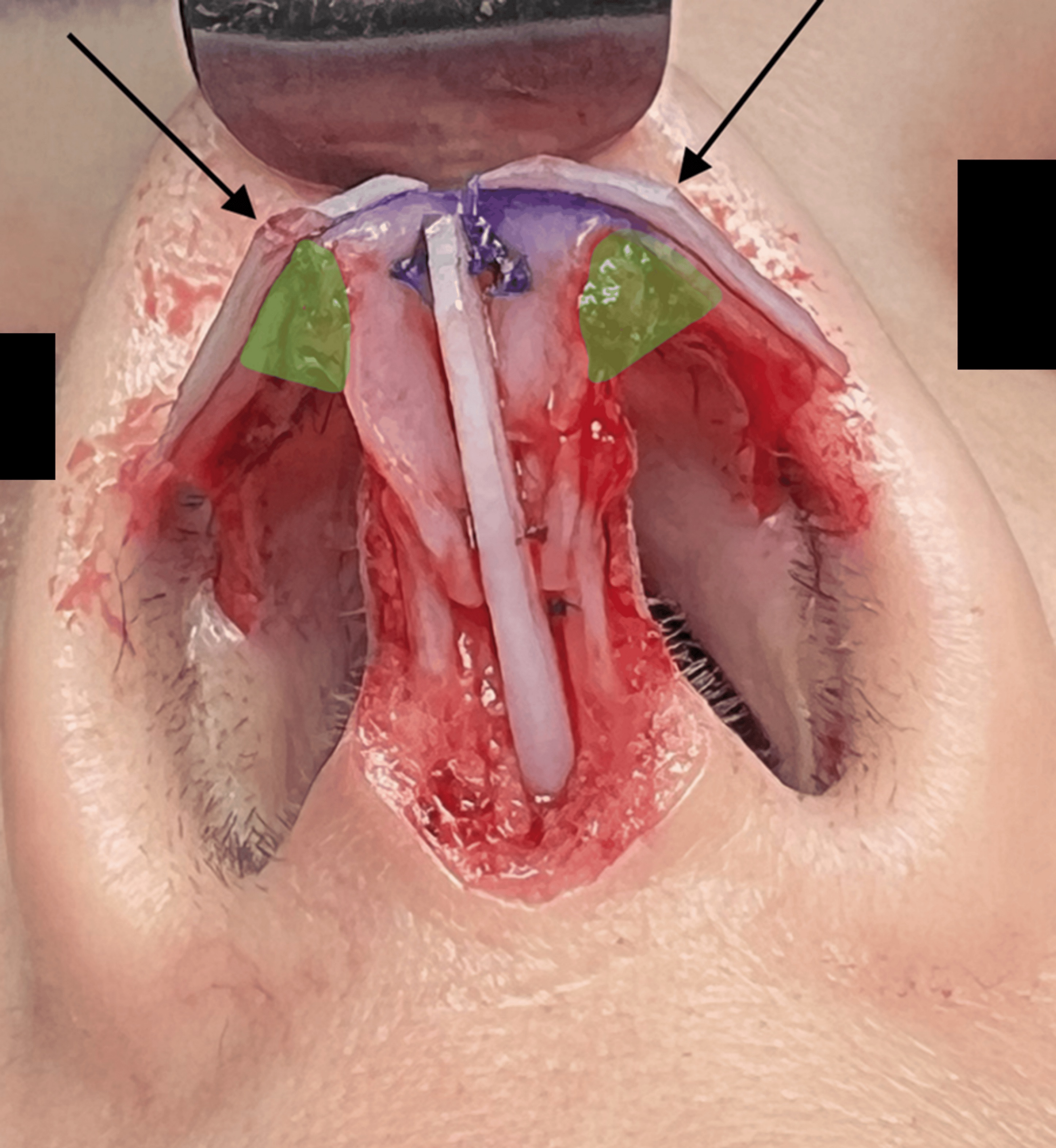
Basal view of the secured modified batten grafts The black arrow indicates the area that will provide the new luminous tip points of the nose. The areas highlighted in green provide structural support to the soft triangle.

The graft is secured using simple interrupted 6-0 polypropylene sutures at the domal level, with at least three sutures distributed along the graft to ensure complete fixation (Figure 3). The graft is anchored to the caudal border of the lateral crus or positioned slightly offset over it, depending on the patient's specific structural needs (Figure 4). Functionally and aesthetically, the graft position can be adjusted in subtle increments. Placing the graft 1-2 mm anterior to the dome may support tip projection and help refine the domal light reflexes, whereas positioning it 1-2 mm anterior to the caudal border of the lateral crus can provide additional structural support to the soft triangle and improve the transition to the nasal tip. 

Final contouring and tip-width refinement

As the final step, the graft vertices contributing to the tip region are refined and beveled, and the edges are smoothed until an approximate tip width of 8 mm is achieved.

Results

**Figure 5:**
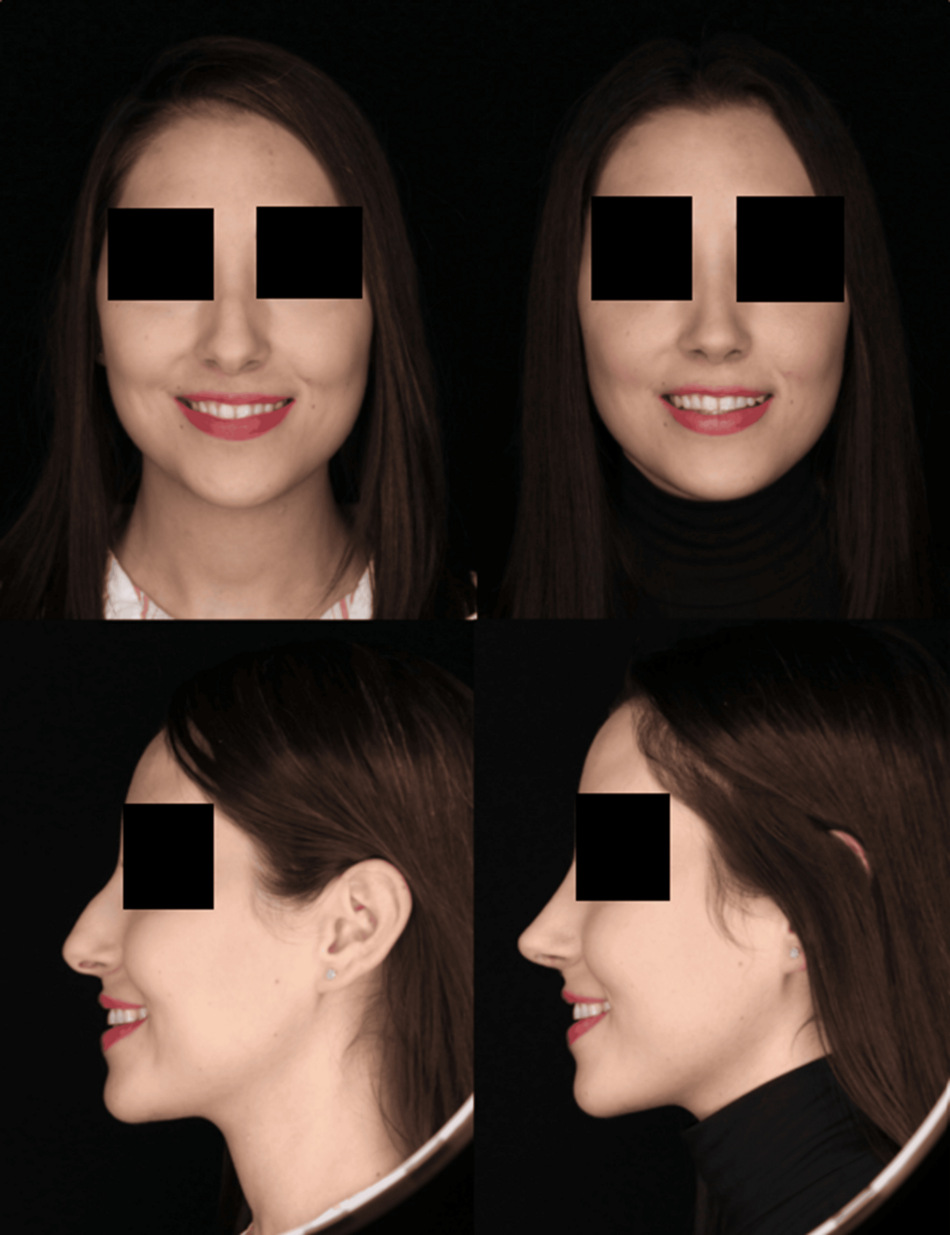
Preoperative (left column) and three-month postoperative (right column) front and lateral views Note: Written informed consent to include these images in an open-access article was obtained from the patient.

**Figure 6:**
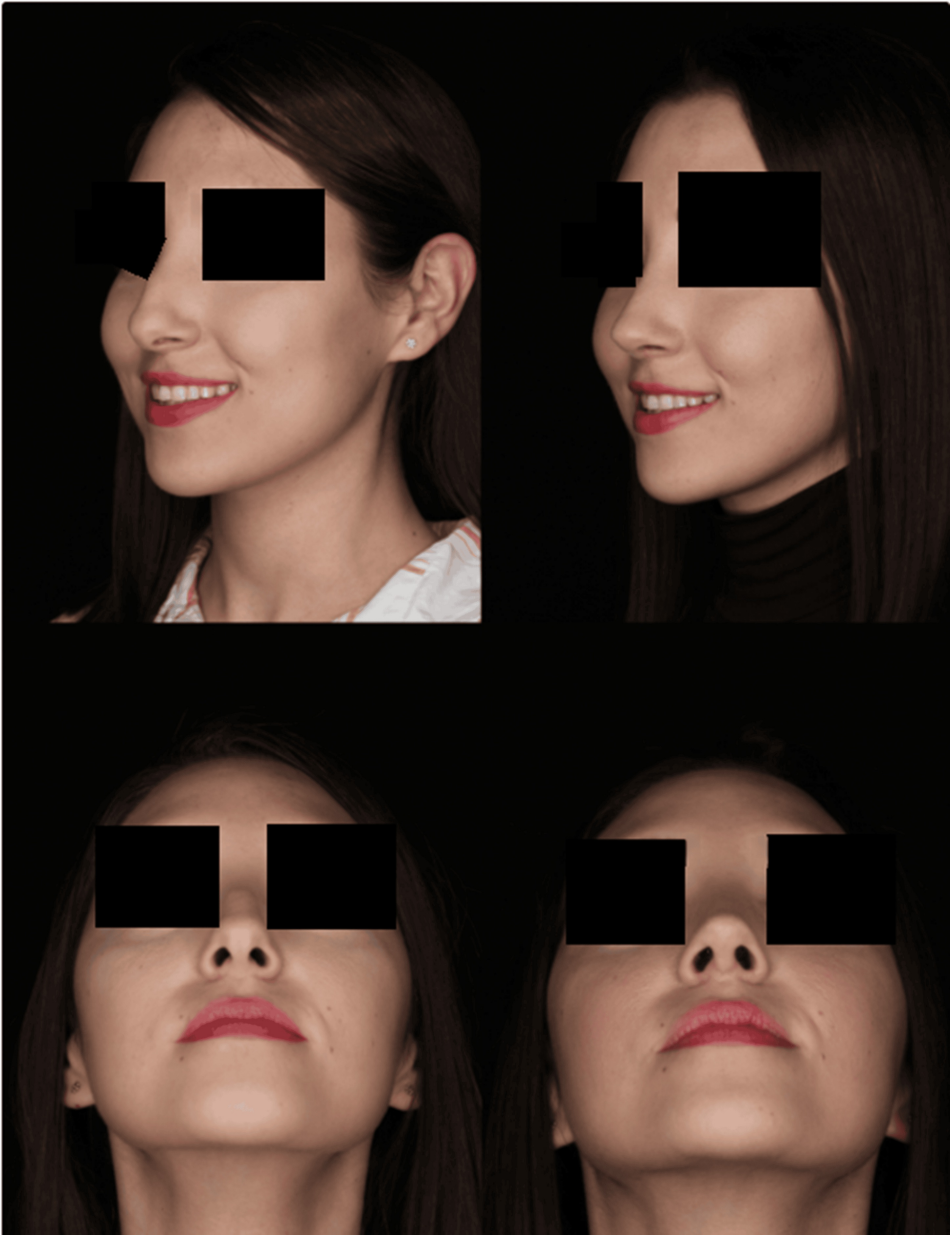
Preoperative (left column) and three-month postoperative (right column) oblique and basal views Note: Written informed consent to include these images in an open-access article was obtained from the patient.

Preoperative lateral and basal views demonstrated mid-third concavity of the lateral crura. At the three-month follow-up, following placement of the modified alar batten graft, restoration of alar-tip continuity, stable soft triangle support, improved nasal tip definition, and improved alar margin position were observed (Figure 5 and Figure 6). The patient reported subjective improvement in nasal airflow, with a postoperative NOSE score of 5 and an ROE score of 87.5. No complications were reported. 

## Discussion

Beyond the associated functional impairment, lateral nasal wall insufficiency may manifest as supra-alar pinching, parenthesis deformity, alar rim retraction, basal asymmetries, and a broad, poorly defined nasal tip [5]. The original graft restores function but is limited in its ability to address alar margin deformities or tip contour irregularities that may accompany lateral nasal wall weakness. Our modified alar batten graft is a useful option for addressing these alterations that would otherwise require additional techniques.

The lateral crural strut graft repositions the lateral crura but may require complementary techniques to reshape the tip [5]. Alar rim grafts support the external valve while smoothing the transition from the nasal tip to the alar lobules using a limited amount of graft material; however, they are technically more challenging and may result in long-term asymmetries [4]. Suture-based techniques are particularly useful for mild lateral crural deformities, but small or weak lateral crura may limit their applicability without additional grafting [6]. This limitation is more frequently encountered in patients with inherently flimsy cartilages, a recurrent finding in the Latino population [7].

The trapezoidal modified alar batten graft, placed as a superstructural overlay over the lateral crura, reinforces the lower lateral cartilages and increases their vertical dimension, simultaneously addressing lateral nasal wall insufficiency and crural deformities. An additional advantage is its ability to modify nasal tip projection when the graft is positioned 1-2 mm anterior to the domes. Placement of the modified graft results in the native dome resting just posterior to the soft triangle. In this configuration, the preserved native dome acts as a stabilizing buttress that protects and reinforces the soft triangle, while the graft provides additional lateral nasal wall support. This restores continuity of the alar-tip unit and is particularly advantageous in patients with soft triangle weakness, significant alar retraction, and loss of alar-tip definition. Adjusting the graft width at the domal level allows modification of the domal light reflexes, while placing it 1-2 mm anterior to the caudal border of the lateral crura provides soft triangle support and improves the transition from the lobule to the nasal tip. The results observed in this case are preliminary, and conclusions regarding the efficacy of this technique compared with existing methods require further validation.

## Conclusions

The modified alar batten graft expands the indications of the traditional alar batten graft by combining lateral nasal wall reinforcement with predictable aesthetic refinement of the nasal tip and alar margins. Acting as a superstructural support over the lateral crura and stabilizing the soft triangle, the technique restores continuity of the alar-tip unit while providing structural support to the external nasal valve. This approach is particularly valuable in patients with thin or weakened lateral crura, soft triangle weakness, and significant alar retraction, for whom isolated grafts or suture techniques may be insufficient. Although encouraging, these findings are preliminary and warrant long-term follow-up in larger patient series.
